# Use of Wearable Devices in Individuals With or at Risk for Cardiovascular Disease in the US, 2019 to 2020

**DOI:** 10.1001/jamanetworkopen.2023.16634

**Published:** 2023-06-07

**Authors:** Lovedeep S. Dhingra, Arya Aminorroaya, Evangelos K. Oikonomou, Arash Aghajani Nargesi, Francis Perry Wilson, Harlan M. Krumholz, Rohan Khera

**Affiliations:** 1 Section of Cardiovascular Medicine, Department of Internal Medicine, Yale School of Medicine, New Haven, Connecticut; 2 Heart and Vascular Center, Brigham and Women’s Hospital, Harvard Medical School, Boston, Massachusetts; 3 Clinical and Translational Research Accelerator, Department of Medicine, Yale School of Medicine, New Haven, Connecticut; 4 Center for Outcomes Research and Evaluation (CORE), Yale New Haven Hospital, New Haven, Connecticut; 5 Department of Health Policy and Management, Yale School of Public Health, New Haven, Connecticut; 6 Section of Health Informatics, Department of Biostatistics, Yale School of Public Health, New Haven, Connecticut

## Abstract

**Importance:**

Wearable devices may be able to improve cardiovascular health, but the current adoption of these devices could be skewed in ways that could exacerbate disparities.

**Objective:**

To assess sociodemographic patterns of use of wearable devices among adults with or at risk for cardiovascular disease (CVD) in the US population in 2019 to 2020.

**Design, Setting, and Participants:**

This population-based cross-sectional study included a nationally representative sample of the US adults from the Health Information National Trends Survey (HINTS). Data were analyzed from June 1 to November 15, 2022.

**Exposures:**

Self-reported CVD (history of heart attack, angina, or congestive heart failure) and CVD risk factors (≥1 risk factor among hypertension, diabetes, obesity, or cigarette smoking).

**Main Outcomes and Measures:**

Self-reported access to wearable devices, frequency of use, and willingness to share health data with clinicians (referred to as *health care providers* in the survey).

**Results:**

Of the overall 9303 HINTS participants representing 247.3 million US adults (mean [SD] age, 48.8 [17.9] years; 51% [95% CI, 49%-53%] women), 933 (10.0%) representing 20.3 million US adults had CVD (mean [SD] age, 62.2 [17.0] years; 43% [95% CI, 37%-49%] women), and 5185 (55.7%) representing 134.9 million US adults were at risk for CVD (mean [SD] age, 51.4 [16.9] years; 43% [95% CI, 37%-49%] women). In nationally weighted assessments, an estimated 3.6 million US adults with CVD (18% [95% CI, 14%-23%]) and 34.5 million at risk for CVD (26% [95% CI, 24%-28%]) used wearable devices compared with an estimated 29% (95% CI, 27%-30%) of the overall US adult population. After accounting for differences in demographic characteristics, cardiovascular risk factor profile, and socioeconomic features, older age (odds ratio [OR], 0.35 [95% CI, 0.26-0.48]), lower educational attainment (OR, 0.35 [95% CI, 0.24-0.52]), and lower household income (OR, 0.42 [95% CI, 0.29-0.60]) were independently associated with lower use of wearable devices in US adults at risk for CVD. Among wearable device users, a smaller proportion of adults with CVD reported using wearable devices every day (38% [95% CI, 26%-50%]) compared with overall (49% [95% CI, 45%-53%]) and at-risk (48% [95% CI, 43%-53%]) populations. Among wearable device users, an estimated 83% (95% CI, 70%-92%) of US adults with CVD and 81% (95% CI, 76%-85%) at risk for CVD favored sharing wearable device data with their clinicians to improve care.

**Conclusions and Relevance:**

Among individuals with or at risk for CVD, fewer than 1 in 4 use wearable devices, with only half of those reporting consistent daily use. As wearable devices emerge as tools that can improve cardiovascular health, the current use patterns could exacerbate disparities unless there are strategies to ensure equitable adoption.

## Key Points

**Question:**

What are the patterns of use of wearable devices among individuals with or at risk for cardiovascular disease (CVD) in the US?

**Findings:**

In this cross-sectional study based on a representative sample of 9303 US adults in 2019 and 2020, 18% with established CVD and 26% at risk for CVD reported using wearable devices compared with 29% of the general population. Older age, lower educational attainment, and lower household income were associated with significantly lower odds of wearable device use, while 82% of all users reported willingness to share their health data with clinicians.

**Meaning:**

These findings suggest that as wearable devices emerge as tools that can improve cardiovascular health, their current use patterns could exacerbate disparities unless there are strategies to ensure equitable adoption.

## Teaser

This nationally representative cross-sectional study of US adults in 2019 to 2020 assesses the use of wearable devices across cardiovascular risk groups and evaluates patterns of use across key demographic and socioeconomic subgroups.

## Introduction

Wearable devices are increasingly being identified as a strategy to improve the detection and management of cardiovascular disease (CVD).^1,2^ Moreover, the availability and use of wearable devices has increased over the past decade, especially for health monitoring during the COVID-19 pandemic.^3,4,5^ These devices, commonly including fitness bands and smartwatches, are electronic devices worn on or around the body, containing specialized sensors for monitoring user physiology.^1,3^ Wearable devices are commonly used for activity, heart rate, and sleep tracking.^2^ More sophisticated wearable devices allow electrocardiographic^6,7,8^ and blood pressure^9^ monitoring and can provide cost-effective methods for screening of cardiac arrhythmias,^10,11^ management of hypertension,^12^ and other clinical and lifestyle interventions.^13^

While regulatory bodies and clinical societies increasingly endorse wearable device use in clinical care,^14,15,16^ we need to evaluate whether current patterns of uptake of these devices would allow their use for improving cardiovascular (CV) care at the national level and the implications of their use for health equity. First, it is critical to identify the demographic patterns of wearable device uptake and potential disparities across sociodemographic subgroups. Second, to evaluate their role as a key technology that supports health care, it is pertinent to assess the consistency of their use and whether users would be willing to share data with their clinicians (referred to as *health care providers* in the survey).^2,17^ There is limited nationally representative information on the users of wearable devices, their use practices, and their interest in sharing this information. Accordingly, in a nationally representative cross-sectional study of US adults in 2019 to 2020, we assessed the use of wearable devices across CV risk groups and evaluated patterns of use across key demographic and socioeconomic subgroups.

## Methods

Our study followed the Strengthening the Reporting of Observational Studies in Epidemiology (STROBE) reporting guideline. The Health Information National Trends Survey (HINTS) protocol has been reviewed by the Westat Institutional Review Board and was exempted from review and informed consent by the US National Institutes of Health Office of Human Subjects Research Protections.^18^ Since our study used publicly available data with deidentified information, it represents nonhuman subject research and is outside the purview of the Yale institutional review board.

### Data Source

In this cross-sectional study, we combined all HINTS participants for 2019 and 2020 to obtain a nationally representative sample for this period. HINTS is the largest nationally representative survey of health technology utilization among community-dwelling adults.^19^ It is designed as a serial cross-sectional survey supported by the National Cancer Institute and collects data on the knowledge, attitudes, and use of cancer- and health-related information.^20^ Comprehensive methodologic reports on HINTS have previously been published.^18^

Briefly, each cycle deployed a 2-stage, stratified, probability sampling strategy. All nonvacant residential addresses of the US in the Marketing Systems Group database were stratified based on the density of racial and ethnic minority populations into areas with high (≥34% of the population are Hispanic or Black) and low levels of minority populations. First, an equal-probability sample of these addresses was selected with oversampling of high-level minority addresses to improve the representativeness of minority populations. Next, 1 adult was selected from the sampled household for participation. Full-sample weights were computed to explicitly account for nonresponse and noncoverage of populations and to enhance the accuracy of the estimates and permit generalizability of the results to the national population.^18^ Further details on the survey are included in the eMethods in Supplement 1.

### Study Population

We included all 9303 adult participants from HINTS 2019 and 2020. We identified individuals with CVD and CVD risk factors based on the survey questionnaire.^21^ We defined the presence of CVD as an affirmative response to the survey question, “Has a doctor or other health professional ever told you that you had any of the following medical conditions: A heart condition such as heart attack, angina or congestive heart failure?” We defined the population at risk for CVD as participants without overt CVD, but with at least 1 CVD risk factor among hypertension, diabetes, obesity, or cigarette smoking. The survey included questions about the presence of hypertension and diabetes.^21^ We defined obesity as a body mass index (calculated as weight in kilograms divided by height in meters squared) of at least 30 and cigarette smoking as currently smoking every day or on some days.

### Study Outcomes

The primary outcome of the study was the proportion of participants who reported using wearable devices to monitor their activity and health over the preceding 12 months. We evaluated national estimates of these proportions across CVD risk groups and across key demographic and socioeconomic subgroups. In participants who reported using wearable devices, we also assessed the frequency of wearable device use and the willingness to share health data with clinicians. Additionally, we evaluated associations of sociodemographic characteristics and clinical features with wearable device use across those with CVD or CVD risk factors as exploratory outcomes.

### Study Covariates

Self-reported demographic and socioeconomic characteristics included age, sex, race and ethnicity, educational attainment, household income, and area of residence. Age was recorded as a continuous measure and was categorized into 18 to 49 years, 50 to 64 years, and 65 years or older. Race and ethnicity consisted of Hispanic, non-Hispanic Black, non-Hispanic White, and others (including non-Hispanic American Indian or Alaska Native, non-Hispanic Asian, non-Hispanic Native Hawaiian or other Pacific Islander, or more than 1 race). Educational attainment was classified as up to high school graduate, college education, and postbaccalaureate degree. We defined annual household income levels as less than $20 000, $20 000 to $50 000, and more than $50 000. Area of residence was categorized into metropolitan and nonmetropolitan (micropolitan, small town, and rural) based on the size and direction of primary commuting flows according to the rural-urban commuting area codes.^18^

### Statistical Analysis

Data were analyzed from June 1 to November 15, 2022. All nationally representative analyses accounted for the complex survey design, including the sampling clusters, stratification by minority population density, and personal-level full weight. The survey χ^2^ test was used to compare categorical sociodemographic variables and CVD risk factors between participants with and without access to wearable devices.

In exploratory analyses, we assessed individual features associated with wearable device use among those with CVD or CVD risk factors. We sequentially evaluated the risk-adjusted use of wearable devices in adults with CVD or CVD risk factors using survey logistic regression models that accounted for (1) demographic characteristics (age, sex, and race and ethnicity), (2) demographic characteristics and CVD risk factors (hypertension, diabetes, obesity, and smoking), and (3) demographic characteristics, CVD risk factors, and socioeconomic status (educational attainment, household income, and area of residence). All statistical tests were 2 sided, with the level of statistical significance set at α = .05. All analyses were performed using R, version 4.2.0 (R Project for Statistical Computing).

## Results

### Population Characteristics

There were 9303 participants in HINTS during 2019 and 2020, representing 247.3 million US adults with a mean (SD) age of 48.8 (17.9) years, including 51% (95% CI, 49%-53%) women. Overall, 933 HINTS participants (10.0%) had CVD, representing an estimated 20.3 million (95% CI, 17.8-22.8 million) adults with CVD in the US, with a mean (SD) age of 62.2 (17.0) years, including 43% (95% CI, 37%-49%) women. There were 5185 participants (55.7%) at risk for CVD, representing 134.9 million (95% CI, 128.3-141.4 million) US adults. The estimated mean (SD) age of the at-risk US adults was 51.4 (16.9) years, with 51% (95% CI, 48%-53%) being women.

### Estimated National Use of Wearable Devices

A total of 9193 HINTS participants (98.8%) responded to the wearable use survey question. Among respondents, 2368 (25.8%) reported using a wearable device for monitoring their health and activity in the preceding 12 months. These participants represent a weighted estimate of 71.5 million (95% CI, 67.1-75.1 million) US adults, or 29% (95% CI, 27%-30%) of all US adults (Table 1). In comparison, 168 HINTS respondents with CVD (18.3%) used wearable devices, representing an estimated 3.6 million (95% CI, 2.7-4.6 million) or 18% (95% CI, 14%-23%) of US adults with CVD. Among respondents at risk for CVD, 1188 (23.2%) used wearable devices, representing an estimated 34.5 million (95% CI, 31.4-37.4 million) US adults, or 26% (95% CI, 24%-28%) of all US adults at risk for CVD (Table 1).

**Table 1.** Overall Use of Wearable Devices

Population	Users of wearable devices, No. (%)	Weighted national estimate (95% CI)	Weighted % (95% CI)
Participants of HINTS, 2019 and 2020	2368/9193 (25.8)	71.5 (67.1-75.1) million	29 (27-30)
Participants with CVD	168/918 (18.3)	3.6 (2.7-4.6) million	18 (14-23)
Participants at risk for CVD	1188/5121 (23.2)	34.5 (31.4-37.4) million	26 (24-28)

Abbreviations: CVD, cardiovascular disease; HINTS, Health Information National Trends Survey.

Compared with individuals without CVD or CVD risk factors, individuals with CVD (odds ratio [OR], 0.52; 95% CI, 0.38-0.71) and those at risk for CVD (OR, 0.73; 95% CI, 0.62-0.87) were significantly less likely to use a wearable device (*P* < .001 for both). However, the lower use of wearable devices in these groups was a function of demographic and socioeconomic differences between these groups, with no significant difference in their use in these populations after adjusting for these differences between individuals with CVD (OR, 0.90; 95% CI, 0.61-1.33) and those with CVD risk factors (OR, 0.95; 95% CI, 0.78-1.16) (eFigure in Supplement 1).

### Demographic Distribution of Wearable Device Use Across CVD Risk Profiles

**Figure 1.**
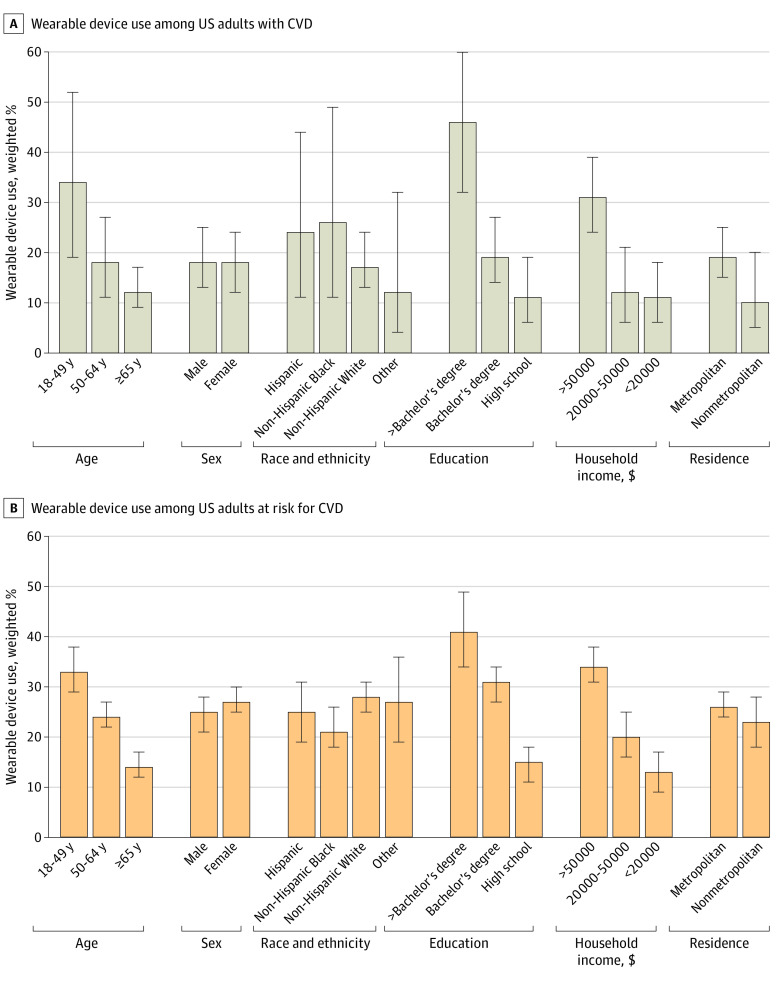
Wearable Device Use Across Demographic and Socioeconomic Subgroups Whiskers indicate 95% CIs. CVD indicates cardiovascular disease.

While adults 65 years or older represent 49% (95% CI, 43%-54%) of all adults with CVD, 12% (95% CI, 9%-17%) of them reported using wearable devices compared with 18% (95% CI, 11%-27%) in the group aged 50 to 64 years and 34% (95% CI, 19%-52%) in the group aged 18 to 49 years. In those at risk for but without established CVD, 14% (95% CI, 12%-17%) of adults 65 years or older used wearable devices compared with 24% (95% CI, 22%-27%) in the group aged 50 to 64 years and 33% (95% CI, 29%-38%) in the group aged 18 to 49 years (Figure 1 and eTables 1 and 2 in Supplement 1).

There were no significant differences in wearable device use between men and women among individuals with CVD (18% [95% CI, 12%-24%] for women vs 18% [95% CI, 13%-25%] for men; *P* = .92) and those at risk for CVD (27% [95% CI, 25%-30%] for women vs 25% [95% CI, 21%-28%] for men; *P* = .20). There were no significant differences in wearable device use by race and ethnicity among those with CVD. However, in the at-risk population, Black individuals were less likely to use wearable devices compared with White individuals (Figure 1 and eTable 2 in Supplement 1).

With respect to educational attainment, among individuals with CVD, those with a postbaccalaureate degree represented 7% (95% CI, 5%-9%) of the population; those with up to a high school education represented 38% (95% CI, 32%-45%) of the population. The use of wearable devices was substantially higher among those with higher educational attainment, representing 46% (95% CI, 32%-60%) with a postbaccalaureate degree and 11% (95% CI, 6%-19%) with up to a high school education. Among adults at risk for CVD, 41% (95% CI, 34%-49%) of those with a postbaccalaureate degree used wearable devices compared with 15% (95% CI, 11%-18%) of those educated up to high school graduation (eTables 1 and 2 in Supplement 1).

With respect to household income, among individuals with CVD, 31% (95% CI, 24%-39%) with household incomes of more than $50 000 used wearable devices compared with 11% (95% CI, 6%-18%) of those with incomes less than $20 000. Similarly, in the at-risk population, 34% (95% CI, 31%-38%) of individuals with higher household incomes used these devices compared with 13% (95% CI, 9%-17%) of individuals with lower household incomes (Figure 1 and eTable 2 in Supplement 1). No significant difference was observed in wearable device use based on area of residence among individuals with CVD and at risk for CVD (Figure 1 and eTable 2 in Supplement 1).

### Wearable Device Use in Individuals With and at Risk for CVD Across Sociodemographic Subgroups

**Figure 2.**
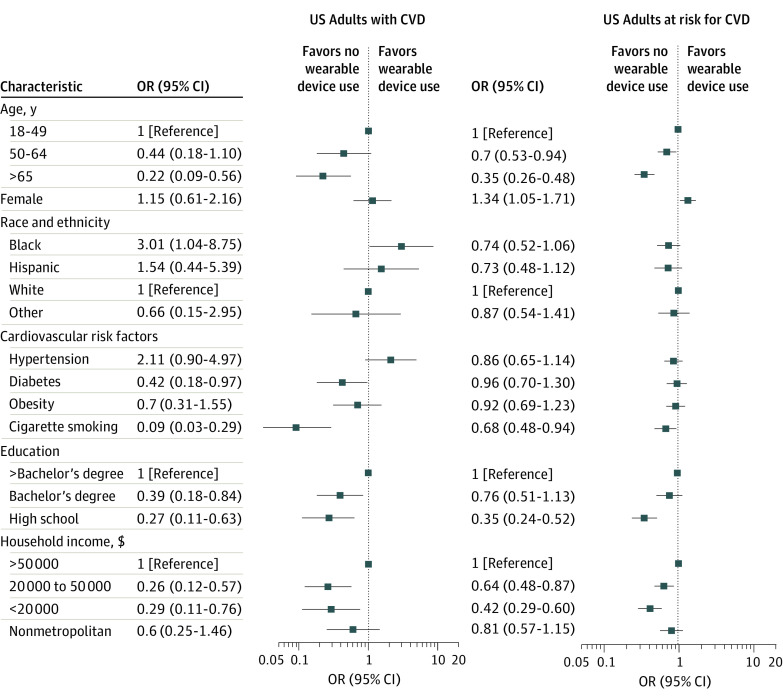
Multivariate Sociodemographic Analysis of Wearable Device Use CVD indicates cardiovascular disease; OR, odds ratio.

After accounting for differences in demographic characteristics, CVD risk factors, and socioeconomic features, individuals in the oldest age group (≥65 years) had only one-fifth the odds of wearable device use in the population with CVD (OR, 0.22 [95% CI, 0.09-0.56]; *P* = .002) and one-third the odds in the at-risk population (OR, 0.35 [95% CI, 0.26-0.48]; *P* < .001) compared with individuals in the group aged 18 to 49 years (eTables 5 and 6 in Supplement 1 and Figure 2). While sex differences in wearable device use were not significant in individuals with CVD (OR for women vs men, 1.15 [95% CI, 0.61-2.16]), women had higher wearable device use among US adults at risk for CVD (OR, 1.34 [95% CI, 1.05-1.71]; *P* = .02) (model 3, eTable 6 in Supplement 1 and Figure 2).

Among those with CVD, non-Hispanic Black individuals did not have significantly different odds of wearable device use compared with other racial and ethnic groups, after accounting for demographic and clinical differences. However, after accounting for socioeconomic differences, Black individuals with CVD had 3-fold higher odds of wearable device use (OR, 3.01 [95% CI, 1.04-8.75]; *P* = .05) compared with White individuals (Figure 2). In the at-risk population, Black individuals at risk for CVD had significantly lower wearable use after accounting for differences in demographic characteristics and CVD risk factors but not after additionally accounting for differences in socioeconomic features of educational attainment, household income, and area of residence (OR, 0.74 [95% CI, 0.52-1.06]) (Figure 2).

Across CVD risk factors, in multivariable assessments, the presence of diabetes, but not hypertension or obesity, was independently associated with lower wearable device use in individuals with CVD (OR, 0.42 [95% CI, 0.18-0.97]; *P* = .04) (model 3, eTable 5 in Supplement 1 and Figure 2). Cigarette smoking was associated with lower odds of wearable device use by over 90% among individuals with CVD (OR, 0.09 [95% CI, 0.03-0.29]; *P* < .001) and over 30% among individuals at risk for CVD (OR, 0.68 [95% CI, 0.48-0.94]; *P* = .02) (model 3, eTables 5 and 6 in Supplement 1 and Figure 2).

After accounting for differences in demographic characteristics, CVD risk factors, and socioeconomic features, lower educational attainment (up to high school graduation) was associated with lower odds of wearable device use in the population with CVD (OR, 0.27 [95% CI, 0.11-0.63]; *P* = .004) and the at-risk population (OR, 0.35 [95% CI, 0.24-0.52]; *P* < .001) compared with the attainment of a postbaccalaureate degree. Similarly, a household income less than $20 000 was associated with less than one-third the odds of wearable device use in US adults with CVD (OR, 0.29 [95% CI, 0.11-0.76]; *P* = .01) and greater than 50% lower odds in individuals at risk for CVD (OR, 0.42 [95% CI, 0.29-0.60]; *P* < .001) compared with a household income of more than $50 000 (eTables 5 and 6 in Supplement 1 and Figure 2). The area of residence was not associated with wearable device use, after adjusting for demographic characteristics, CVD risk factors, and socioeconomic features (Figure 2).

### Frequency of Wearable Device Use

Of all the US adults using wearable devices, an estimated 49% (95% CI, 45%-53%) reported using wearable devices every day. This was lower among adults with CVD, with 38% (95% CI, 26%-50%) reporting using their devices every day compared with 48% (95% CI, 43%-53%) among individuals at risk for CVD.

**Figure 3.**
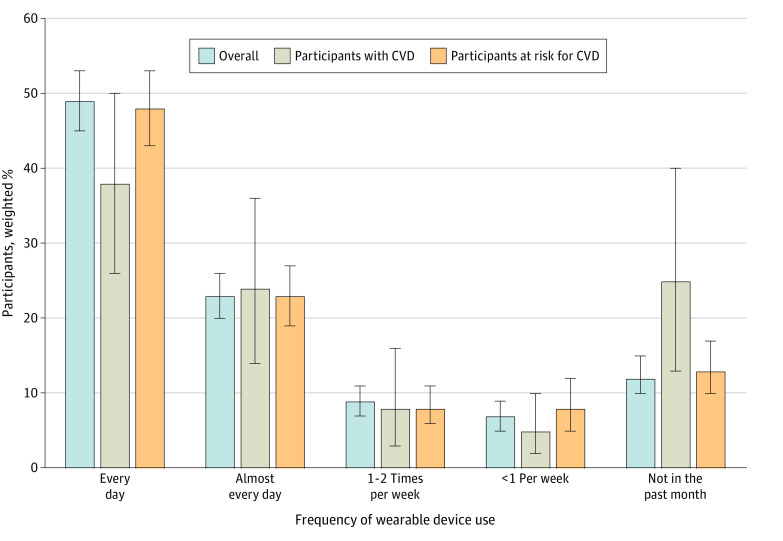
National Estimates of Frequency of Use of Wearable Devices Whiskers indicate 95% CIs. CVD indicates cardiovascular disease.

On the other end, an estimated 12% (95% CI, 10%-15%) of all US adults using wearable devices had not used them in the preceding month. In contrast, among individuals with access to wearable devices, 25% (95% CI, 13%-40%) of all individuals with CVD and 13% (95% CI, 10%-17%) of all at-risk individuals reported not having used their devices in the preceding month (Figure 3 and eTable 3 in Supplement 1).

### Willingness to Share Health Data

Of all US adults using wearable devices across CVD risk profiles, most reported willingness to share their wearable health data with their physician. This corresponds to 82% (95% CI, 79%-84%) of all US adults using wearable devices, representing 57.5 million (95% CI, 53.6-61.5 million) US adults. Similarly, 83% (95% CI, 70%-92%) of individuals with CVD, representing 2.9 million (95% CI, 2.1-3.8 million) US adults, and 81% (95% CI, 76%-85%) of adults at risk for CVD and using wearable devices, representing 27.3 million (95% CI, 24.5-30.1 million) US adults, expressed willingness to share their wearable health data (Table 2). There were no significant differences across key demographic, clinical, or socioeconomic subgroups considering willingness to share wearable health data with clinicians (eTable 7 in Supplement 1).

**Table 2.** Willingness to Share Wearable Data Among Participants Using Wearable Devices

Willingness to share data with clinicians by population	
Participants of HINTS, 2019 and 2020 (n = 2368)	
No. (%)	1905 (80.4)
Weighted No. (95% CI)	57.5 million (53.6-61.5 million)
Weighted % (95% CI)	82 (79-84)
Participants with CVD (n = 164)	
No. (%)	137 (83.5)
Weighted No. (95% CI)	2.9 million (2.1-3.8 million)
Weighted % (95% CI)	83 (70-92)
Participants at risk for CVD (n = 1172)	
No. (%)	945 (80.6)
Weighted No. (95% CI)	27.3 million (24.5-30.1 million)
Weighted % (95% CI)	81 (76-85)

Abbreviations: CVD, cardiovascular disease; HINTS, Health Information National Trends Survey.

## Discussion

In this nationally representative study of contemporary patterns of wearable device use among US adults, we estimated that 72 million, or one-third of the US adults, used wearable devices, and the use was significantly lower among those with CVD, with only 18% reporting wearable use. Being 65 years or older, having no more than a high school education, and having a low household income were independently associated with a substantially lower use of wearable devices. Moreover, one-quarter of the adults with CVD who used wearable devices reported not using their device in the preceding month, which was nearly twice the respective rate for the at-risk and the overall populations. Of note, wearable devices have the potential for CV health management, as most users of wearable devices across sociodemographic and CVD risk groups reported willingness to share health data with their clinicians.

There has been a lack of national data on the use of wearable devices among individuals with CVD and CVD risk factors, despite a broad recognition of their expanding role in tracking CV health. The devices can potentially improve CV health through monitoring and promoting activity, early detection of arrhythmias, access to portable 1-lead electrocardiograms, and a suite of emerging features for CV health.^2,9,22,23,24,25,26,27^ The present study finds that, in an era with expanding use of wearable devices,^9,28,29^ these devices are substantially underused among those with CVD compared with the general US adult population. These differences suggest that populations who stand to gain the most from the use of innovative technologies for CVD management are the least likely to use them. Moreover, the less frequent use of wearable devices among individuals with lower socioeconomic status and educational attainment may represent a missed opportunity in expanding care in CVD^10,11,30^ for these vulnerable groups that have historically also had significantly lower access to such preventive care.^31,32^

Key barriers to the broader use of these devices are likely their cost and their technological accessibility for older individuals.^33^ Our study supports both as potential mechanisms through demonstrated lower use in individuals from low-income households and older adults. Moreover, most of these devices are individually purchased, despite the availability of technology relevant for disease monitoring.^9,11^ A potential strategy to broaden the use of these devices may be their coverage by health insurance providers, but any further use would require rigorous evidence that unequivocally establishes their role in disease management.^34,35^ Moreover, the lower use of wearable devices among patients with lower educational attainment and older adults also suggests a need for adapting user interface for utility as well as dedicated training on their role in health management.^36^ Finally, a dedicated study of barriers to access and use of these devices is essential to ensure their equitable uptake.^33^

A key observation is that 1 in 4 individuals with CVD who owned a wearable device did not use their device in the preceding month. This suggests that wearable device ownership is insufficient to drive their potential health benefits.^37^ Among wearable device users, social incentive–based gamification strategies that have been proposed to promote use of the devices^2,24,30,38^ likely require empirical evaluation for their impact on behavioral patterns. Moreover, the infrequent use of the devices among owners with CVD also has implications for future studies, as compliance-promoting initiatives would be essential to integrate into interventions using wearable devices.

### Limitations

Our study has several limitations. First, the study is based on self-report, with the potential for misclassification on both populations with CVD and wearable use patterns. However, the study instruments are validated and conducted as a part of a large National Institutes of Health study that ensured the best available approach to address these questions.^18,39^ Second, we are limited by the ability to study key CV disorders, as the study questionnaire assessed several key populations with CVD together. Therefore, populations with atrial fibrillation and other arrhythmias, who may derive a larger benefit, could not be uniquely assessed. Furthermore, despite differences in health features, the specific type of wearable device used could not be assessed. Third, the response rates for HINTS were about one-third, which could produce a nonresponse bias. However, the response rates for HINTS are similar to those of other population-based surveys with similar methods,^40^ and the survey weights account for participant nonresponse.^18^ Fourth, following the COVID-19 pandemic, the patterns of wearable device use for health monitoring may change. However, nationally representative contemporary data on the use of wearable devices are not available yet. It may be essential to assess how these patterns of use evolve as more recent data become available.

## Conclusions

Among individuals with or at risk for CVD, fewer than 1 in 4 use wearable devices, with only half of those reporting consistent daily use. If wearable devices emerge as tools that can improve CV health, then the current use patterns could exacerbate disparities unless there are strategies to ensure equitable adoption.

## Notes

Supplement 1. **eMethods.** Details About Health Information National Trends Survey (HINTS) 2019 and 2020 **eFigure.** Use of Wearables Among US Adults With Cardiovascular Disease or Cardiovascular Risk Factors Before and After Adjustment for Sociodemographic Differences **eTable 1.** Demographic Characteristics, Cardiovascular Disease, and Cardiovascular Risk Factors in Health Information National Trends Survey Respondents, 2019 and 2020 **eTable 2.** Demographic Distribution of Wearable Use in Individuals With or at Risk of Cardiovascular Disease **eTable 3.** Frequency of Use in Participants Using Wearable Devices **eTable 4.** Univariable Demographic Predictors of Wearable Device Use in Individuals With Cardiovascular Disease **eTable 5.** Multivariable Models for Wearable Device Use in Individuals With Cardiovascular Disease **eTable 6.** Multivariable Models for Wearable Device Use in Individuals at Risk of Cardiovascular Disease **eTable 7.** Distribution of Demographic Characteristics, Cardiovascular Risk Factors, and Socioeconomic Features Based on Willingness to Share Wearable Device Heath Data With Clinicians in Participants With or at Risk of Cardiovascular Disease

Supplement 2.
Data Sharing Statement
