# Supplementary material for: Use of Wearable Devices in Individuals With or at Risk for Cardiovascular Disease in the US, 2019 to 2020
Source: JAMA Netw Open. 2023 Jun 7;6(6):e2316634. doi: 10.1001/jamanetworkopen.2023.16634 (PMC10248745; doi:10.1001/jamanetworkopen.2023.16634)
Supplement: Supplement 1. — eMethods. Details About Health Information National Trends Survey (HINTS) 2019 and 2020 eFigure. Use of Wearables Among US Adults With Cardiovascular Disease or Cardiovascular Risk Factors Before and After Adjustment for Sociodemographic Differences eTable 1. Demographic Characteristics, Cardiovascular Disease, and Cardiovascular Risk Factors in Health Information National Trends Survey Respondents, 2019 and 2020 eTable 2. Demographic Distribution of Wearable Use in Individuals With or at Risk of Cardiovascular Disease eTable 3. Frequency of Use in Participants Using Wearable Devices eTable 4. Univariable Demographic Predictors of Wearable Device Use in Individuals With Cardiovascular Disease eTable 5. Multivariable Models for Wearable Device Use in Individuals With Cardiovascular Disease eTable 6. Multivariable Models for Wearable Device Use in Individuals at Risk of Cardiovascular Disease eTable 7. Distribution of Demographic Characteristics, Cardiovascular Risk Factors, and Socioeconomic Features Based on Willingness to Share Wearable Device Heath Data With Clinicians in Participants With or at Risk of Cardiovascular Disease [file jamanetwopen-e2316634-s001.pdf]

Supplementary Online Content

Dhingra LS, Aminorroaya A, Oikonomou EK, et al. Use of wearable devices in individuals with or at risk for cardiovascular disease in the US, 2019 to 2020. *JAMA Netw Open*. 2023;6(6):e2316634. doi:10.1001/jamanetworkopen.2023.16634

eMethods. Details About Health Information National Trends Survey (HINTS) 2019 and 2020

eFigure. Use of Wearables Among US Adults With Cardiovascular Disease or Cardiovascular Risk Factors Before and After Adjustment for Sociodemographic Differences

eTable 1. Demographic Characteristics, Cardiovascular Disease, and Cardiovascular Risk Factors in Health Information National Trends Survey Respondents, 2019 and 2020

eTable 2. Demographic Distribution of Wearable Use in Individuals With or at Risk of Cardiovascular Disease

eTable 3. Frequency of Use in Participants Using Wearable Devices

eTable 4. Univariable Demographic Predictors of Wearable Device Use in Individuals With Cardiovascular Disease

eTable 5. Multivariable Models for Wearable Device Use in Individuals With Cardiovascular Disease

eTable 6. Multivariable Models for Wearable Device Use in Individuals at Risk of Cardiovascular Disease

eTable 7. Distribution of Demographic Characteristics, Cardiovascular Risk Factors, and Socioeconomic Features Based on Willingness to Share Wearable Device Heath Data With Clinicians in Participants With or at Risk of Cardiovascular Disease

This supplementary material has been provided by the authors to give readers additional information about their work.

© 2023 Dhingra LS et al. *JAMA Network Open*.

eMethods. Details About Health Information National Trends Survey (HINTS) 2019 and 2020

Cycle 3 of HINTS 5 was conducted from January to April 2019, via an online web pilot survey and via mail-in surveys. The response rates for the paper-only mail-in survey and the web pilot survey were 30.2% and 30.6%, respectively. HINTS 5 Cycle 4 was conducted from February to June 2020, via a paper-only mail-in survey. The response rate for HINTS 5 Cycle 4 was 36.7%.

Accounting for patterns of historically observed nonresponse and noncoverage, HINTS used data from the American Community Survey conducted by the US Census Bureau and the National Health Interview Survey to calibrate the selection weights for participants. Each HINTS participant received a set of one full-sample weight and 50 replicate weights. The full-sample weights were computed by calculating household- and individual-level initial weights, accounting for historically observed patterns of non-response and calibrating individual-level weights to population counts. The replicate weights were computed according to the 'delete one' jackknife replication method. Because HINTS is conducted with a random sample of adults and weighted to the US population, these survey methods allow for inference of nationally representative estimates.

From 2019 onwards, HINTS included the following questions about the use of wearable devices for health monitoring:

- WearableDevTrackHealth: B9. In the past 12 months, have you used an electronic wearable device to monitor or track your health or activity? For example, a Fitbit, AppleWatch or Garmin Vivofit...

**WearableDevTrackHealth**	**Value Label**
-9	Missing data (Not Ascertained)
-7	Missing data (Web partial - Question Never Seen)
1	Yes
2	No

- FreqWearDevTrackHealth: B10. In the past month, how often did you use a wearable device to track your health?

**FreqWearDevTrackHealth**	**Value Label**
-9	Missing data (Not Ascertained)
-7	Missing data (Web partial - Question Never Seen)
-6	Missing data (Filter Missing)
-5	Multiple responses selected in error
-2	Question answered in error (Commission Error)
-1	Inapplicable, coded 1 in WearableDevTrackHealth
1	Everyday
2	Almost everyday
3	1-2 times per week
4	Less than once per week
5	I did not use a wearable device in the past month

- WillingShareData_HCP: B11a. Would you be willing to share health data from your wearable device with your health care provider?

**WillingShareData_HCP**	**Value Label**
-9	Missing data (Not Ascertained)
-7	Missing data (Web partial - Question Never Seen)
-6	Missing data (Filter Missing)
-2	Question answered in error (Commission Error)
-1	Inapplicable, coded 1 in WearableDevTrackHealth
1	Yes
2	No

© 2023 Dhingra LS et al. *JAMA Network Open*.

eFigure. Use of Wearables Among US Adults With Cardiovascular Disease or Cardiovascular Risk Factors Before and After Adjustment for Sociodemographic Differences

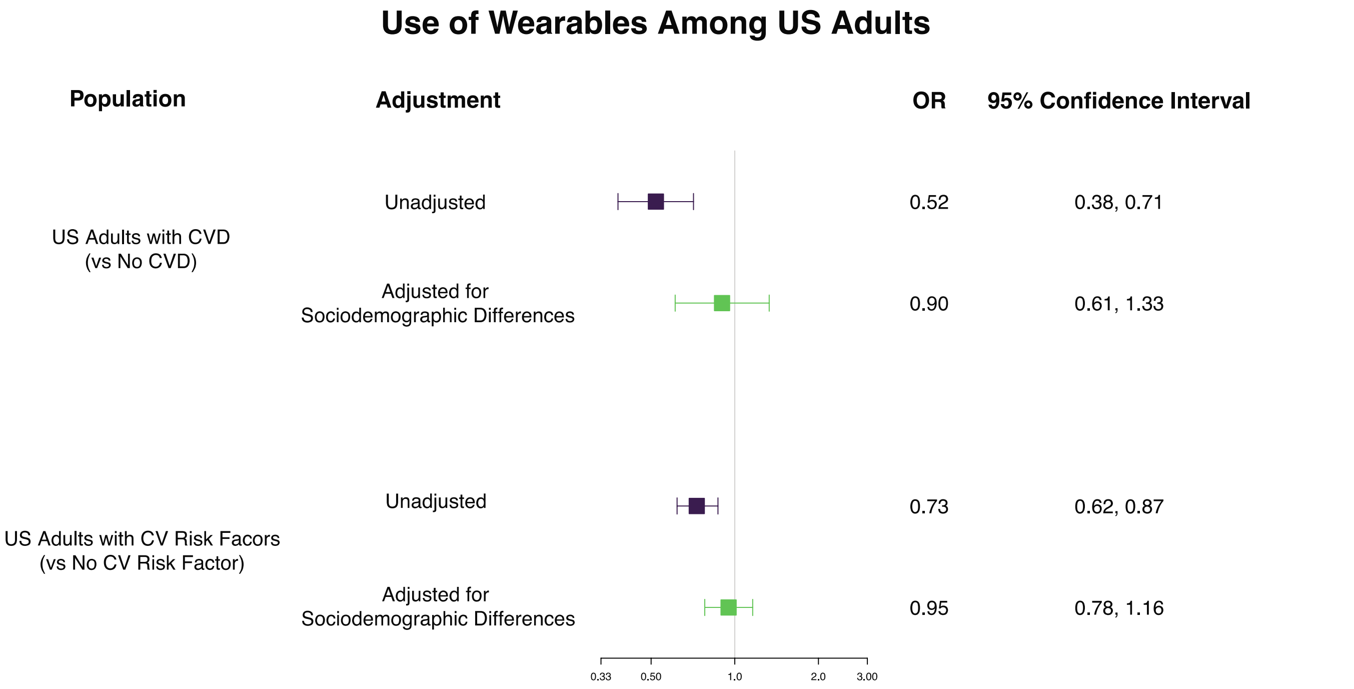

Abbreviations: CV, Cardiovascular; CVD, Cardiovascular Disease; OR, Odds Ratio; US, United States

© 2023 Dhingra LS et al. *JAMA Network Open*.

eTable 1. Demographic Characteristics, Cardiovascular Disease, and Cardiovascular Risk Factors in Health Information National Trends Survey Respondents, 2019 and 2020

**Characteristic**	**Participants of HINTS, 2019 and 2020 (N=9303)**		**Participants with CVD (N=933)**		**Participants at risk of CVD (N=5185)**	
	**Number (%)**	**Weighted Percentage (95% CI)**	**Number (%)**	**Weighted Percentage (95% CI)**	**Number (%)**	**Weighted Percentage (95% CI)**
**Demographics**						
**Age Group**						
18-49	2864 (31.4%)	50% (48% to 52%)	72 (7.82%)	17% (13% to 23%)	1292 (25.3%)	45% (42% to 47%)
50-64	2829 (31.0%)	29% (28% to 31%)	228 (24.8%)	34% (20% to 40%)	1750 (34.3%)	33% (31% to 35%)
>65	3431 (37.6%)	21% (19% to 22%)	621 (67.4%)	49% (43% to 54%)	2066 (40.4%)	23% (21% to 24%)
**Sex**						
Male	3797 (41.8%)	49% (47% to 51%)	487 (53.3%)	57% (51% to 63%)	2122 (41.6%)	49% (47% to 52%)
Female	5277 (58.2%)	51% (49% to 53%)	427 (46.7%)	43% (37% to 49%)	2976 (58.4%)	51% (48% to 53%)
**Race and Ethnicity**						
Non-Hispanic White	5187 (62.2%)	63% (62% to 65%)	530 (67.5%)	71% (65% to 76%)	2771 (59.3%)	62% (60% to 64%)
Non-Hispanic Black	1158 (13.9%)	11% (10% to 12%)	103 (13.1%)	10% (7% to 14%)	807 (17.3%)	14% (12% to 15%)
Hispanic	1326 (15.9%)	17% (16% to 18%)	104 (13.2%)	12% (8% to 17%)	742 (15.9%)	17% (15% to 19%)
Other	669 (8.0%)	8% (7% to 9%)	48 (6.11%)	7% (4% to 11%)	353 (7.55%)	7% (6% to 9%)
**Cardiovascular Risk Factors**						
**CVD**	933 (10.2%)	8% (7% to 9%)	933 (100%)	-	0 (0%)	-
**Any Cardiovascular Risk Factor**	6042 (67.0%)	62% (60% to 64%)	818 (88.2%)	89% (86% to 91%)	5185 (100%)	-
**Hypertension**	4088 (44.9%)	36% (34% to 38%)	723 (78.5%)	76% (71% to 80%)	3344 (64.7%)	55% (53% to 58%)
**Diabetes Mellitus**	1974 (21.7%)	18% (16% to 19%)	406 (44.3%)	43% (37% to 48%)	1561 (30.4%)	26% (24% to 28%)
**Obesity**	3044 (33.8%)	34% (32% to 36%)	390 (42.7%)	44% (38% to %50)	2643 (51.8%)	56% (53% to 59%)
**Cigarette Smoking**	1053 (11.5%)	13% (12% to 15%)	122 (13.4%)	17% (12% to 22%)	916 (17.9%)	22% (20% to 24%)
**Socioeconomic Factors**						
**Education**						
Post-Baccalaureate Degree	1692 (18.8%)	12% (11% to 13%)	113 (12.5%)	7% (5% to 9%)	825 (16.3%)	10% (9% to 11%)
College Education	5053 (56.1%)	58% (56% to 60%)	466 (51.7%)	55% (48% to 62%)	2781 (55.0%)	55% (52% to 57%)

© 2023 Dhingra LS et al. *JAMA Network Open*.

Up to High School Graduate	2258 (25.1%)	31% (29% to 32%)	322 (35.7%)	38% (32% to 45%)	1452 (28.7%)	35% (33% to 38%)
**Household Income**						
>$50,000	4563 (55.3%)	59% (57% to 61%)	321 (39.2%)	37% (31% to 43%)	2364 (51.2%)	55% (53% to 57%)
$20,000 to $50,000	2155 (26.1%)	24% (23% to 26%)	269 (32.8%)	41% (34% to 48%)	1297 (28.1%)	25% (23% to 28%)
<$20,000	1528 (18.5%)	17% (15% to 18%)	229 (28.0%)	22% (18% to 27%)	959 (20.8%)	19% (18% to 21%)
**Residence**						
Metropolitan	8163 (87.8%)	86% (85% to 87%)	801 (85.9%)	86% (82% to 89%)	4502 (86.8%)	84% (82% to 86%)
Non-Metropolitan	1139 (12.2%)	14% (13% to 15%)	132 (14.1%)	14% (11% to 18%)	683 (13.2%)	16% (14% to 18%)

Abbreviations: CI, Confidence Intervals; CVD, Cardiovascular Disease; HINTS, Health Information National Trends Survey

© 2023 Dhingra LS et al. *JAMA Network Open*.

eTable 2. Demographic Distribution of Wearable Use in Individuals With or at Risk of Cardiovascular Disease

Characteristic	Individuals with CVD (N=933)			Individuals at risk of CVD (N=5185)		
	Number (%)^	Weighted Percentage[a] (95% CI)	P Value	Number (%)^	Weighted Percentage[a] (95% CI)	P Value
**Demographics**						
**Age Group**						
18-49	30 (41.7%)	34% (19% to 52%)	0.03	462 (35.8%)	33% (29% to 38%)	<0.001
50-64	42 (18.7%)	18% (11% to 27%)		433 (25.0%)	24% (22% to 27%)	
>65	92 (15.2%)	12% (9% to 17%)		282 (13.9%)	14% (12% to 17%)	
**Sex**						
Male	80 (16.8%)	18% (13% to 25%)	0.92	437 (20.8%)	25% (21% to 28%)	0.20
Female	82 (19.5%)	18% (12% to 24%)		737 (25.0%)	27% (25% to 30%)	
**Race and Ethnicity**						
Non-Hispanic White	100 (19.1%)	17% (13% to 24%)	0.53	683 (24.9%)	28% (25% to 31%)	0.05
Non-Hispanic Black	21 (20.6%)	26% (11% to 49%)		179 (22.3%)	21% (18% to 26%)	
Hispanic	15 (14.4%)	24% (11% to 44%)		161 (21.9%)	25% (19% to 31%)	
Other	8 (17.4%)	12% (4% to 32%)		99 (28.4%)	27% (19% to 36%)	
**Cardiovascular Risk Factors**						
**Hypertension**	124 (17.4%)	18% (13% to 24%)	0.78	695 (21.0%)	23% (21% to 26%)	0.007
**Diabetes Mellitus**	67 (16.8%)	13% (8% to 19%)	0.02	292 (18.9%)	22% (19% to 26%)	0.04
**Obesity**	73 (18.9%)	17% (11% to 25%)	0.60	658 (25.2%)	28% (25% to 31%)	0.09
**Cigarette Smoking**	12 (10.3%)	5% (2% to 10%)	<0.001	149 (16.4%)	21% (17% to 26%)	0.02

© 2023 Dhingra LS et al. *JAMA Network Open*.

**Socioeconomic Factors**						
**Education**						
Post-Baccalaureate Degree	33 (29.7%)	46% (32% to 60%)	<0.001	289 (35.3%)	41% (34% to 49%)	<0.001
College Education	90 (19.5%)	19% (14% to 27%)		719 (26.1%)	31% (27% to 34%)	
Up to High School Graduate	36 (11.5%)	11% (6% to 19%)		165 (11.5%)	15% (11% to 18%)	
**Household Income**						
>$50,000	86 (26.9%)	31% (24% to 39%)	<0.001	800 (34.2%)	34% (31% to 38%)	<0.001
$20,000 to $50,000	39 (14.9%)	12% (6% to 21%)		209 (16.3%)	20% (16% to 25%)	
<$20,000	25 (11.1%)	11% (6% to 18%)		84 (8.9%)	13% (9% to 17%)	
**Residence**						
Metropolitan	151 (19.2%)	19% (15% to 25%)	0.05	1071 (24.0%)	26% (24% to 29%)	0.21
Non-Metropolitan	17 (13.1%)	10% (5% to 20%)		117 (17.3%)	23% (18% to 28%)	
[a] Percentages represent row percentages (Percentage of participants reporting wearable use)						

Abbreviations: CI, Confidence Intervals; CVD, Cardiovascular Disease; HINTS, Health Information National Trends Survey

© 2023 Dhingra LS et al. *JAMA Network Open*.

eTable 3. Frequency of Use in Participants Using Wearable Devices

Frequency of Wearable Use	Participants of HINTS, 2019 and 2020 (N=2368)		Participants with CVD (N=168)		Participants at risk of CVD (N=1188)	
	**Number (%)**	**Weighted Percentage (95% CI)**	**Number (%)**	**Weighted Percentage (95% CI)**	**Number (%)**	**Weighted Percentage (95% CI)**
Everyday	1148 (49.0%)	49% (45% to 53%)	70 (41.9%)	38% (26% to 50%)	586 (50.0%)	48% (43% to 53%)
Almost Everyday	499 (21.3%)	23% (20% to 26%)	33 (19.8%)	24% (14% to 36%)	244 (20.8%)	23% (19% to 27%)
1-2 times per week	218 (9.31%)	9% (7% to 11%)	13 (7.78%)	8% (3% to 16%)	97 (8.28%)	8% (6% to 11%)
Less than once per week	174 (7.43%)	7% (5% to 9%)	15 (8.98%)	5% (2% to 10%)	91 (7.76%)	8% (5% to 12%)
Not in the last month	303 (12.9%)	12% (10% to 15%)	36 (21.6%)	25% (13% to 40%)	154 (13.1%)	13% (10% to 17%)

Abbreviations: CI, Confidence Intervals; CVD, Cardiovascular Disease; HINTS, Health Information National Trends Survey

© 2023 Dhingra LS et al. *JAMA Network Open*.

eTable 4. Univariable Demographic Predictors of Wearable Device Use in Individuals With Cardiovascular Disease

Characteristic	Individuals with CVD		Individuals at risk of CVD	
	Odds Ratio	P-Value	Odds Ratio	P-Value
**Demographics**				
**Age Group**				
18-49	Reference		Reference	
50-64	0.42 (0.17-1.06)	0.07	0.65 (0.51-0.84)	0.001
>65	0.28 (0.13-0.61)	0.002	0.34 (0.27-0.42)	<0.001
**Sex**				
Male	Reference		Reference	
Female	0.97 (0.55-1.71)	0.92	1.16 (0.92-1.46)	0.20
**Race and Ethnicity**				
Non-Hispanic White	Reference		Reference	
Non-Hispanic Black	1.65 (0.58-4.69)	0.35	0.68 (0.52-0.89)	0.007
Hispanic	1.48 (0.55-3.97)	0.44	0.83 (0.58-1.19)	0.32
Other	0.66 (0.18-2.43)	0.53	0.93 (0.61-1.43)	0.75
**Cardiovascular Risk Factors**				
**Hypertension**	0.91 (0.48-1.74)	0.78	0.75 (0.61-0.92)	0.007
**Diabetes Mellitus**	0.52 (0.30-0.92)	0.03	0.76 (0.59-0.99)	0.04
**Obesity**	0.86 (0.48-1.53)	0.61	1.24 (0.97-1.57)	0.09
**Cigarette Smoking**	0.19 (0.09-0.41)	<0.001	0.73 (0.55-0.97)	0.03
**Socioeconomic Factors**				
**Education**				
Post-Baccalaureate Degree	Reference		Reference	
College Education	0.29 (0.14-0.57)	0.001	0.63 (0.44-0.89)	0.01
Up to High School Graduate	0.14 (0.06-0.31)	<0.001	0.24 (0.17-0.35)	<0.001
**Household Income**				
>$50,000	Reference		Reference	
$20,000 to $50,000	0.29 (0.14-0.63)	0.002	0.47 (0.35-0.63)	<0.001
<$20,000	0.27 (0.14-0.53)	<0.001	0.28 (0.19-0.41)	<0.001
**Residence**				
Metropolitan	Reference		Reference	
Non-Metropolitan	0.49 (0.22-1.09)	0.08	0.81 (0.58-1.14)	0.24

Abbreviations: CI, Confidence Intervals; CVD, Cardiovascular Disease; HINTS, Health Information National Trends Survey

© 2023 Dhingra LS et al. *JAMA Network Open*.

eTable 5. Multivariable Models for Wearable Device Use in Individuals With Cardiovascular Disease

Characteristic	Model 1[a]		Model 2[b]		Model 3[c]	
	Odds Ratio (95% CI)	P-Value	Odds Ratio (95% CI)	P-Value	Odds Ratio (95% CI)	P-Value
**Demographics**						
**Age Group**						
18-49	Reference					
50-64	0.42 (0.16, 1.09)	0.08	0.62 (0.23, 1.69)	0.30	0.44 (0.18, 1.10)	0.08
>65	0.27 (0.12, 0.63)	0.003	0.26 (0.09, 0.72)	0.01	0.22 (0.09, 0.56)	0.002
**Sex**						
Male	Reference					
Female	0.84 (0.46, 1.53)	0.60	0.95 (0.54, 1.69)	0.90	1.15 (0.61, 2.16)	0.70
**Race and Ethnicity**						
Non-Hispanic White	Reference					
Non-Hispanic Black	1.42 (0.49, 4.06)	0.50	2.19 (0.78, 6.13)	0.14	3.01 (1.04, 8.75)	0.05
Hispanic	1.05 (0.41, 2.67)	>0.9	1.13 (0.40, 3.19)	0.80	1.54 (0.44, 5.39)	0.50
Other	0.46 (0.11, 1.89)	0.30	0.80 (0.18, 3.65)	0.80	0.66 (0.15, 2.95)	0.60
**Cardiovascular Risk Factors**						
**Hypertension**	—	—	1.35 (0.53, 3.42)	0.50	2.11 (0.90, 4.97)	0.09
**Diabetes Mellitus**	—	—	0.43 (0.22, 0.86)	0.02	0.42 (0.18, 0.97)	0.04
**Obesity**	—	—	0.72 (0.32, 1.61)	0.40	0.70 (0.31, 1.55)	0.40
**Cigarette Smoking**	—	—	0.13 (0.04, 0.40)	<0.001	0.09 (0.03, 0.29)	<0.001
**Socioeconomic Factors**						
**Education**						
Post-Baccalaureate Degree	—	—	—	—	Reference	
College Education	—	—	—	—	0.39 (0.18, 0.84)	0.02
Up to High School Graduate	—	—	—	—	0.27 (0.11, 0.63)	0.004

© 2023 Dhingra LS et al. *JAMA Network Open*.

**Household Income**						
>$50,000	—	—	—	—	Reference	
$20,000 to $50,000	—	—	—	—	0.26 (0.12, 0.57)	0.001
<$20,000	—	—	—	—	0.29 (0.11, 0.76)	0.01
**Residence**						
Metropolitan	—	—	—	—	Reference	
Non-Metropolitan	—	—	—	—	0.60 (0.25, 1.46)	0.30

[a] Model 1 represents a survey-weighted, multivariable logistic regression model adjusting for demographic characteristics (age, sex, and race and ethnicity).
[b] Model 2 represents a survey-weighted, multivariable logistic regression model adjusting for demographic characteristics and cardiovascular risk factors (hypertension, diabetes mellitus, obesity, cigarette smoking)
[c] Model 3 represents a survey-weighted, multivariable logistic regression model adjusting for demographic characteristics, cardiovascular risk factors, and socioeconomic features (education, household income, and residence)

Abbreviations: CI, Confidence Intervals; CVD, Cardiovascular Disease; HINTS, Health Information National Trends Survey

© 2023 Dhingra LS et al. *JAMA Network Open*.

eTable 6. Multivariable Models for Wearable Device Use in Individuals at Risk of Cardiovascular Disease

Characteristic	Model 1[a]		Model 2[b]		Model 3[c]	
	Odds Ratio (95% CI)	P-Value	Odds Ratio (95% CI)	P-Value	Odds Ratio (95% CI)	P-Value
**Demographics**						
**Age Group**						
18-49	Reference					
50-64	0.66 (0.51, 0.85)	0.002	0.66 (0.50, 0.86)	0.003	0.70 (0.53, 0.94)	0.02
>65	0.32 (0.25, 0.40)	<0.001	0.30 (0.22, 0.40)	<0.001	0.35 (0.26, 0.48)	<0.001
**Sex**						
Male	Reference					
Female	1.26 (0.99, 1.60)	0.06	1.23 (0.97, 1.55)	0.10	1.34 (1.05, 1.71)	0.02
**Race and Ethnicity**						
Non-Hispanic White	Reference					
Non-Hispanic Black	0.60 (0.45, 0.79)	<0.001	0.61 (0.45, 0.81)	0.001	0.74 (0.52, 1.06)	0.10
Hispanic	0.70 (0.48, 1.03)	0.07	0.67 (0.46, 0.99)	0.05	0.73 (0.48, 1.12)	0.20
Other	0.81 (0.53, 1.26)	0.40	0.83 (0.53, 1.31)	0.40	0.87 (0.54, 1.41)	0.60
**Cardiovascular Risk Factors**						
**Hypertension**	—	—	0.93 (0.71, 1.24)	0.60	0.86 (0.65, 1.14)	0.30
**Diabetes Mellitus**	—	—	0.89 (0.67, 1.18)	0.40	0.96 (0.70, 1.30)	0.80
**Obesity**	—	—	0.87 (0.66, 1.16)	0.40	0.92 (0.69, 1.23)	0.60
**Cigarette Smoking**	—	—	0.57 (0.41, 0.78)	<0.001	0.68 (0.48, 0.94)	0.02
**Socioeconomic Factors**						
**Education**						
Post-Baccalaureate Degree	—	—	—	—	Reference	
College Education	—	—	—	—	0.76 (0.51, 1.13)	0.20
Up to High School Graduate	—	—	—	—	0.35 (0.24, 0.52)	<0.001

© 2023 Dhingra LS et al. *JAMA Network Open*.

**Household Income**						
>$50,000	—	—	—	—	Reference	
$20,000 to $50,000	—	—	—	—	0.64 (0.48, 0.87)	0.005
<$20,000	—	—	—	—	0.42 (0.29, 0.60)	<0.001
**Residence**						
Metropolitan	—	—	—	—	Reference	
Non-Metropolitan	—	—	—	—	0.81 (0.57, 1.15)	0.20

[a] Model 1 represents a survey-weighted, multivariable logistic regression model adjusting for demographic characteristics (age, sex, and race and ethnicity).
[b] Model 2 represents a survey-weighted, multivariable logistic regression model adjusting for demographic characteristics and cardiovascular risk factors (hypertension, diabetes mellitus, obesity, cigarette smoking)
[c] Model 3 represents a survey-weighted, multivariable logistic regression model adjusting for demographic characteristics, cardiovascular risk factors, and socioeconomic features (education, household income, and residence)

Abbreviations: CI, Confidence Intervals; HINTS, Health Information National Trends Survey

© 2023 Dhingra LS et al. *JAMA Network Open*.

eTable 7. Distribution of Demographic Characteristics, Cardiovascular Risk Factors, and Socioeconomic Features Based on Willingness to Share Wearable Device Heath Data With Clinicians in Participants With or at Risk of Cardiovascular Disease

Characteristic	Participants with CVD			Participants at risk of CVD		
	Not Willing (N=27)	Willing (N=137)	P-Value	Not Willing (N=227)	Willing (N=945)	P-Value
**Demographics**						
**Age Group**			0.62			0.89
18-49	4 (13.3%)	26 (86.7%)		82 (17.9%)	377 (82.1%)	
50-64	9 (21.4%)	33 (78.6%)		92 (21.5%)	335 (78.5%)	
>65	13 (14.6%)	76 (85.4%)		48 (17.4%)	228 (82.6%)	
**Sex**			0.61			0.24
Male	12 (15%)	68 (85%)		75 (17.4%)	357 (82.6%)	
Female	15 (19%)	64 (81%)		148 (20.4%)	579 (79.6%)	
**Race and Ethnicity**			0.36			0.47
Non-Hispanic White	15 (15.3%)	83 (84.7%)		117 (17.3%)	559 (82.7%)	
Non-Hispanic Black	1 (4.8%)	20 (95.2%)		28 (15.9%)	148 (84.1%)	
Hispanic	2 (13.3%)	13 (86.7%)		36 (22.6%)	123 (77.4%)	
Other	3 (37.5%)	5 (62.5%)		26 (26.5%)	72 (73.5%)	
**Cardiovascular Risk Factors**						
**Hypertension**			0.96			0.31
No	9 (22.5%)	31 (77.5%)		105 (21.7%)	379 (78.3%)	
Yes	18 (14.8%)	104 (85.2%)		122 (17.8%)	565 (82.2%)	
**Diabetes Mellitus**			0.69			0.74
No	15 (15.5%)	82 (84.5%)		171 (19.4%)	709 (80.6%)	
Yes	12 (18.5%)	53 (81.5%)		56 (19.4%)	232 (80.6%)	
**Obesity**			0.33			0.51
No	14 (15.6%)	76 (84.4%)		104 (20.6%)	402 (79.4%)	
Yes	12 (16.7%)	60 (83.3%)		118 (18.1%)	534 (81.9%)	
**Cigarette Smoking**			0.74			0.85
No	25 (16.8%)	124 (83.2%)		189 (18.6%)	827 (81.4%)	
Yes	1 (9.1%)	10 (90.9%)		34 (23%)	114 (77%)	
**Socioeconomic Factors**						
**Education**			0.81			0.10
Post-Baccalaureate Degree	2 (6.2%)	30 (93.8%)		55 (19.2%)	232 (80.8%)	
College Education	15 (16.9%)	74 (83.1%)		124 (17.4%)	587 (82.6%)	
Up to High School Graduate	6 (17.1%)	29 (82.9%)		44 (27.5%)	116 (72.5%)	

© 2023 Dhingra LS et al. *JAMA Network Open*.

**Household Income**			0.67			0.39
>$50,000	13 (15.1%)	73 (84.9%)		135 (17%)	658 (83%)	
$20,000 to $50,000	5 (12.8%)	34 (87.2%)		52 (25.1%)	155 (74.9%)	
<$20,000	4 (17.4%)	19 (82.6%)		14 (17.3%)	67 (82.7%)	
**Residence**			0.08			0.73
Metropolitan	25 (17%)	122 (83%)		204 (19.3%)	852 (80.7%)	
Non-Metropolitan	2 (11.8%)	15 (88.2%)		23 (19.8%)	93 (80.2%)	

Abbreviations: CI, Confidence Intervals; CVD, Cardiovascular Disease

© 2023 Dhingra LS et al. *JAMA Network Open*.
