# Supplementary material for: Use of Wearable Devices in Individuals With or at Risk for Cardiovascular Disease in the US, 2019 to 2020
Source: JAMA Netw Open. 2023 Jun 7;6(6):e2316634. doi: 10.1001/jamanetworkopen.2023.16634 (PMC10248745; doi:10.1001/jamanetworkopen.2023.16634)
Supplement: Supplement 2. — Data Sharing Statement [file jamanetwopen-e2316634-s002.pdf]

Data Sharing Statement

Dhingra. Use of Wearable Devices in Individuals With or at Risk for Cardiovascular Disease in the US, 2019 to 2020. *JAMA Netw Open*. Published June 07, 2023.
doi:10.1001/jamanetworkopen.2023.16634

Data
Data available: Yes
Data types: Deidentified participant data
How to access data: The data are available from the HINTS survey from NCHS directly and our code will be made available
When available: With publication

Supporting Documents
Document types: Statistical/analytic code
How to access documents: rohan.khera@yale.edu
When available: With publication

Additional Information
Who can access the data: Researchers whose proposed use of the data has been approved
Types of analyses: Data will be available from NCHS directly - available for public use
Mechanisms of data availability: Data will be available from NCHS directly - available for public use
Any additional restrictions: None
